# Editorial: Post-transcriptional regulation of embryonic and adult myogenesis

**DOI:** 10.3389/fcell.2022.1063934

**Published:** 2022-10-26

**Authors:** De-Li Shi, Raphaëlle Grifone, Ming Shao

**Affiliations:** ^1^ Developmental Biology Laboratory, CNRS-UMR7622, Institut de Biologie Paris-Seine (IBPS), Sorbonne University, Paris, France; ^2^ Shandong Provincial Key Laboratory of Animal Cell and Developmental Biology, School of Life Sciences, Shandong University, Qingdao, China

**Keywords:** muscle development, regeneration, satellite cells, muscular disorder, RNA-binding proteins, non-coding RNAs, RNA modification

Skeletal muscle is the most abundant tissue in adult vertebrates. It originates from mononucleated myogenic precursor cells that are transformed into multinucleated functional myofibers by a temporally and spatially elaborated regulatory program. Considerable progress has been made in delineating myogenic pathways that contribute to muscle development. Myogenic transcription factors that integrate intrinsic and extrinsic inputs to trigger myogenesis in the embryo have been extensively characterized during the past decades. However, post-transcriptional regulation, such as mRNA processing, localization, stability, polyadenylation and translation, has been less documented in skeletal muscle biology. Therefore, the identification and functional assessment of key players involved in this regulatory paradigm, including but not limited to RNA-binding proteins (RBPs) and non-coding RNAs, are essential for understanding molecular mechanisms controlling myogenic lineage differentiation during embryonic development. In adult life, post-transcriptional regulation of gene expression is also critically required for maintaining tissue homeostasis and plasticity. The remarkable regenerative capacity of adult muscle through activation of muscle stem cells or satellite cells is generally thought to recapitulate embryonic muscle development. Defects in the post-transcriptional regulation of muscle developmental genes are responsible for a large number of congenital abnormalities in humans. It is intriguing to understand how the post-transcriptional mechanisms regulating embryonic myogenesis function to modulate myogenic differentiation of satellite cells and other “non-muscle” stem cells. This Research Topic has collected review and research articles that presented different aspects of post-transcriptional regulation in muscle development, regeneration, and disease.

The formation of skeletal muscle results from a complex interplay of factors coordinating gene expression at different levels. Myostatin acts as an inhibitor of muscle growth. Chen et al. reviewed aspects of its self-regulation and discussed its function in myogenic differentiation and myofiber type conversion. Myostatin not only regulates the synthesis and degradation of muscle-specific proteins, but also induces reactive oxygen species and oxidative stress in skeletal muscle. Understanding the mechanisms by which it regulates myogenesis could have an impact on animal breeding. A comprehensive review by Shi and Grifone outlined current advances in characterizing RBPs-mediated post-transcriptional regulation in muscle development, regeneration and disease. This study detailed the implication of RBPs in myoblast proliferation and differentiation under physiological and pathological conditions. It also emphasized on the biochemical and functional interactions of RBPs in the control of gene expression for myogenic differentiation as well as stem cell quiescence or activation. It is now well established that dysfunctions of many RBPs are either directly or indirectly linked to various muscle disorders or neuromuscular diseases, making them as potential therapeutic targets.

Increasing evidence indicates that non-coding RNAs are also important post-transcriptional regulators and involved in muscle development. Several research articles have provided mechanistic insights into the function of circRNAs and lncRNAs in myogenesis. Using chick skeletal muscle development as a model, Wei et al. reported the role of muscle-enriched *circFNDC3AL* in myogenesis. Mechanistically, *circFNDC3AL* binds to *miR-204* and prevents its inhibitory activity on the expression of B-cell CLL/lymphoma 9 (BCL9) protein that can promote muscle satellite cell proliferation and differentiation. Similarly, Shen et al. found that *circITSN2* is highly expressed in skeletal muscle and induces myogenesis by targeting *miR-2018-5p* to allow the expression of LIM domain protein 7 (LMO7). These works have identified circRNAs as miRNA sponges during muscle development. In another study, Lv et al. examined possible roles of single nucleotide polymorphisms (SNPs) in porcine *lncMGPF* gene, which functions as a positive regulator of muscle differentiation, growth and regeneration. They provided evidence for a potential contribution of several SNPs to meat production traits through regulation of *lncMGPF* stability and activity in muscle development.

Further illustrating the implication of non-coding RNAs in myogenesis, a research article by Singh et al. analyzed miRNAs-mediated gene expression in muscle differentiation. This work showed that cyclase-associated protein 1 (CAP1), which functions as a critical regulator of actin treadmilling, plays a role in cytoskeletal remodeling during myogenic differentiation. Its timely down-regulation is necessary for proper myotube formation. Several muscle-enriched miRNAs, including *miR-1*, *miR-133*, and *miR-206*, have conserved binding sites at the 3′-UTR of *Cap1* mRNA. They post-transcriptionnally inhibit CAP1 expression to promote myoblast fusion and muscle formation.

It is now well established that RNA modifications play important roles in the transcriptional and post-transcriptional regulation of gene expression. N^6^-methyladenosine (m^6^A) RNA methylation is the most prevalent modification within eukaryotic mRNAs and non-coding RNAs. A research article by Xie et al. examined how members of the m^6^A core methyltransferase complex regulate muscle differentiation and regeneration. This study showed that down-regulation of METTL3/14 expression promotes differentiation of C2C12 and primary mouse skeletal muscle cells. The authors identified *MNK2* mRNA, which encodes a known regulator of ERK/MAPK signaling, as an YTHDF1-dependent target of METTL3/14-mediated m^6^A methylation. The results suggest that METTL3/14 inhibit myogenic differentiation through activation of ERK/MAPK signaling. However, the METTL3/14-MNK2 axis was activated following acute skeletal muscle injury, implying that it may function at early stages of muscle regeneration to promote activation and proliferation of muscle stem cells. Yu et al. thoroughly reviewed m^6^A methylation modification in myogenic differentiation. This work presented m^6^A enzyme system and detailed regulatory roles of m^6^A modulators during embryonic myogenesis and postnatal muscle development, including myotube formation, skeletal muscle homeostasis, regeneration and hypertrophic response. The review also discussed functional interactions between m^6^A modulators and miRNAs by proposing additional modes of post-transcriptional regulation in skeletal myogenesis.

In summary, the aim of this Research Topic was to provide insights into molecular mechanisms that operate in muscle cells. Discussions in review articles and novel data presented in research articles integrated several aspects of current advances in studying the post-transcriptional gene regulation during muscle development, regeneration and disease. They not only contribute to better understand muscle formation under physiological and pathological conditions, but also help to uncover molecular pathways underlying the muscle repair process that presents significant implications in regenerative medicine.

